# Intracellular Regulome Variability Along the Organ of Corti: Evidence, Approaches, Challenges, and Perspective

**DOI:** 10.3389/fgene.2018.00156

**Published:** 2018-05-08

**Authors:** Kevin T. Booth, Hela Azaiez, Israt Jahan, Richard J. H. Smith, Bernd Fritzsch

**Affiliations:** ^1^Molecular Otolaryngology and Renal Research Laboratories, Department of Otolaryngology, University of Iowa, Iowa City, IA, United States; ^2^Interdisciplinary Graduate Program in Molecular Medicine, Carver College of Medicine, University of Iowa, Iowa City, IA, United States; ^3^Department of Biology, University of Iowa, Iowa City, IA, United States

**Keywords:** hair cells, auditory, cell type specificity, trasncriptomics, non-coding RNA, hair cell restoration

## Abstract

The mammalian hearing organ is a regular array of two types of hair cells (HCs) surrounded by six types of supporting cells. Along the tonotopic axis, this conserved radial array of cell types shows longitudinal variations to enhance the tuning properties of basilar membrane. We present the current evidence supporting the hypothesis that quantitative local variations in gene expression profiles are responsible for local cell responses to global gene manipulations. With the advent of next generation sequencing and the unprecedented array of technologies offering high throughput analyses at the single cell level, transcriptomics will become a common tool to enhance our understanding of the inner ear. We provide an overview of the approaches and landmark studies undertaken to date to analyze single cell variations in the organ of Corti and discuss the current limitations. We next provide an overview of the complexity of known regulatory mechanisms in the inner ear. These mechanisms are tightly regulated temporally and spatially at the transcription, RNA-splicing, mRNA-regulation, and translation levels. Understanding the intricacies of regulatory mechanisms at play in the inner ear will require the use of complementary approaches, and most probably, a combinatorial strategy coupling transcriptomics, proteomics, and epigenomics technologies. We highlight how these data, in conjunction with recent insights into molecular cell transformation, can advance attempts to restore lost hair cells.

## Introduction

**Figure 1:**
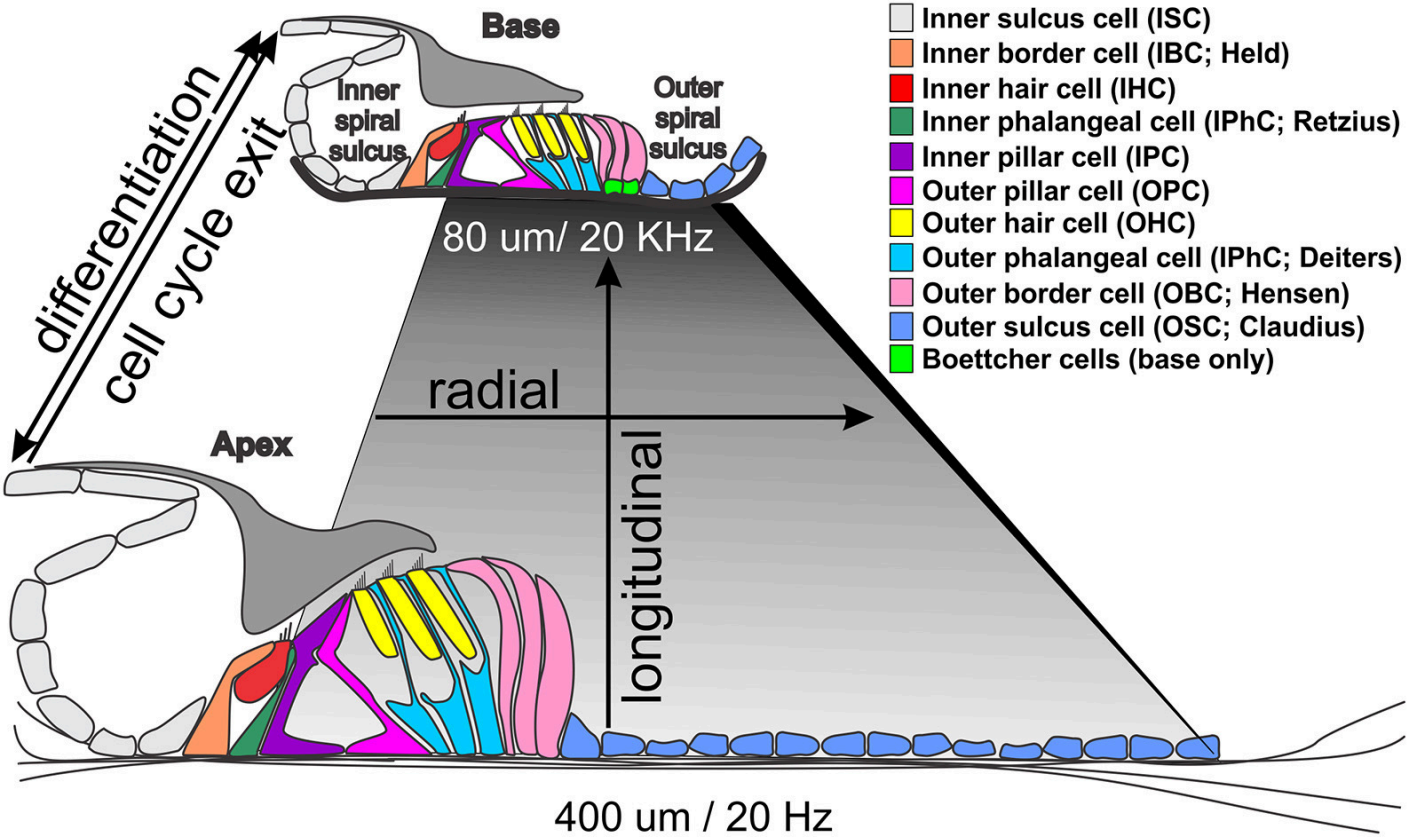
Morphological differences between the base and apex of the ~35 mm long human organ of Corti. Note that the basilar membrane (shaded rhombus) is shorter and thicker at the high frequency base (darker gray) and wider and thinner at the low frequency apex (light gray). The only cellular difference are Boettcher cells in the vassal turn but all cells and stereocilia are longer in the apex. Temporal differences in cell cycle exit (apex to base from 12 to 14 embryonic days in mice) differ from differentiation progression indicated by Atoh1 upregulation (from ~14 embryonic to postnatal day 1) that may drive local variations in gene expression profiles to enhance tuning properties of the basilar membrane through local cell size variation.

Organ development typically requires a cellular resolution of gene expression whereby diffusible factors regulate overall gene expression (Gierer and Meinhardt, 1972; Meinhardt, 2015), that is reinforced through local interactions via delta-notch neighboring cell interactions (Sato et al., 2016; Koon et al., 2017) to regulate local quantitative variations of gene expression profiles. This interplay establishes both distinct cell types as well as functionally significant variations in gene expression profiles of cellular phenotypes. The organ of Corti, the mammalian hearing organ, is one such system that has to establish local variations of several distinct cell types (Groves et al., 2013) (Figure 1). This local variation of a common cellular theme enables mechanisms to enhance frequency tuning properties of the basilar membrane in an apparently smooth progression from base to apex (Richter et al., 2007). In essence, the organ of Corti provides both a highly stereotyped cellular configuration of two types of mechanosensory hair cells (HCs) surrounded by six distinct supporting cell types, each with unique radial distribution (Jahan et al., 2015a; Munnamalai and Fekete, 2016) and systematic longitudinal (apex to base) local (neighboring cells within the same region of the Organ of Corti) variations (Figure 1). While past research has established the functional significance of different mechanotransducting HC types, more recent work has demonstrated that even apparently minor local variations may result in deafness (Tan et al., 2018). Ultimately, how global cell type specification and local variation are regulated must be understood for successful regeneration of HCs as a rehabilitation option for deafness (Sha et al., 2001). This review provides an overview of the cellular architecture of the organ of Corti and its local variation, describes the techniques in use to identify expression profiles, highlights limitations in our understanding of functional regulation of genes and proteins, and outlines the technical advances needed to collect relevant single-cell expression profiles to guide restoration of a lost organ of Corti. We conclude with examples of changes in expression from developmental time points to the mature organ of Corti to highlight the complex cellular mosaicism of the inner ear.

## Organization of the adult mammalian organ of corti

The mammalian organ of Corti is a stereotyped assembly of cells in the radial dimension (from the center or modiolus to the lateral wall) that varies in length to enable hearing at species-specific frequencies that extend from the infrasonic in elephants to the ultrasonic in bats and dolphins (Figure 1). Radially, there are two anatomically distinct compartments, an inner compartment around the inner hair cells (IHCs) and an outer compartment around the outer hair cells (OHCs) (Held, 1926; Pujol and Lavigne-Rebillard, 1992). In addition, there is graded variation of each cell type along the length of the basilar membrane to the reticular lamina, which we will refer to as longitudinal organization (Figure 1): All cells are shorter near the base and longer near the apex, with matching stereocilia length to enhance local basilar membrane tuning properties (Lewis et al., 1985). Radially, the inner compartment consists of (from medial to lateral) inner border cells (IBCs), IHCs, inner phalangeal cells (IPhCs), and inner pillar cells (IPCs). The outer compartment consists of outer pillar cells (OPCs), three rows of OHCs, three rows of outer phalangeal cells (OPhCs; aka Deiters' cells) and 2–4 rows of outer border cells (OBCs; aka Henson cells; Boettcher cells near the base). The organ of Corti is bounded laterally by outer sulcus cells (OSCs; aka Claudius cells) laterally and medially by inner sulcus cells (ISCs).

The two types of HCs and six distinct types of supporting cells have apical and basal discrete cellular contacts. For example, the base of an IHC is in contact only with afferents, whereas it has extensive lateral contact with neighboring IHCs and surrounding IBCs and IPhCs near its apex. In addition, the necks of IHCs are in contact with IBCs and IPhCs, however at the reticular lamina the IHCs are in contact with the IPCs. Select supporting cells form a regular mosaic at specific levels with HCs, while at other levels, a different pattern of cellular interaction arises. For example, in one plane OPhCs and OHCs form a mosaic, however near the basilar membrane OPhCs are in broad contact with each other without an intervening cell type. That a regular mosaic of HCs/supporting cells is present only in certain areas of the organ of Corti and at certain levels between the reticular lamina and the basilar membrane suggests that in addition to delta/notch inhibition, a complex interplay of many factors is required to define and regulate specific cell types at specific radial locations (Jahan et al., 2015a; Munnamalai and Fekete, 2016).

Variations of this common theme are the length changes of cells and/or processes along the cochlea. For example, IHCs are longer in the apex as compared to the base, and have longer stereocilia to reinforce the mechanical properties of the basilar membrane (short and stiff in the base, wide and more pliable in the apex; Figure 1). The shorter stereocilia in the base change the micromechanical properties between the much stiffer basilar membrane and the differently shaped tectorial membrane to enhance high frequency hearing. To accommodate the increased width of the basilar membrane at the apex, there is an expansion of the pillar cells with a wider tunnel of Corti, which radially stretches the organ of Corti without changing the overall cell type distribution. While regional (apex, middle, or base) variations are hypothesized to enhance frequency-specific hearing (Kielczynski, 2017), data are mostly correlative. To date, for example, no studies have changed local HC variation to make basal HCs as long as their apical counterparts, or vice versa, in part because we are only beginning to understand the mechanisms of this local variation (Ciganović et al., 2017). Longitudinal HC variations seems to translate a temporal variation of differentiation, starting with *Atoh1* expression near the base, relative to cell cycle exit, starting at the apex, to generate spatial cell-type variations (Kopecky et al., 2013; Yizhar-Barnea and Avraham, 2017).

## Genetic manipulations reveal local variations in expression profiles leading to differential effects

**Figure 2:**
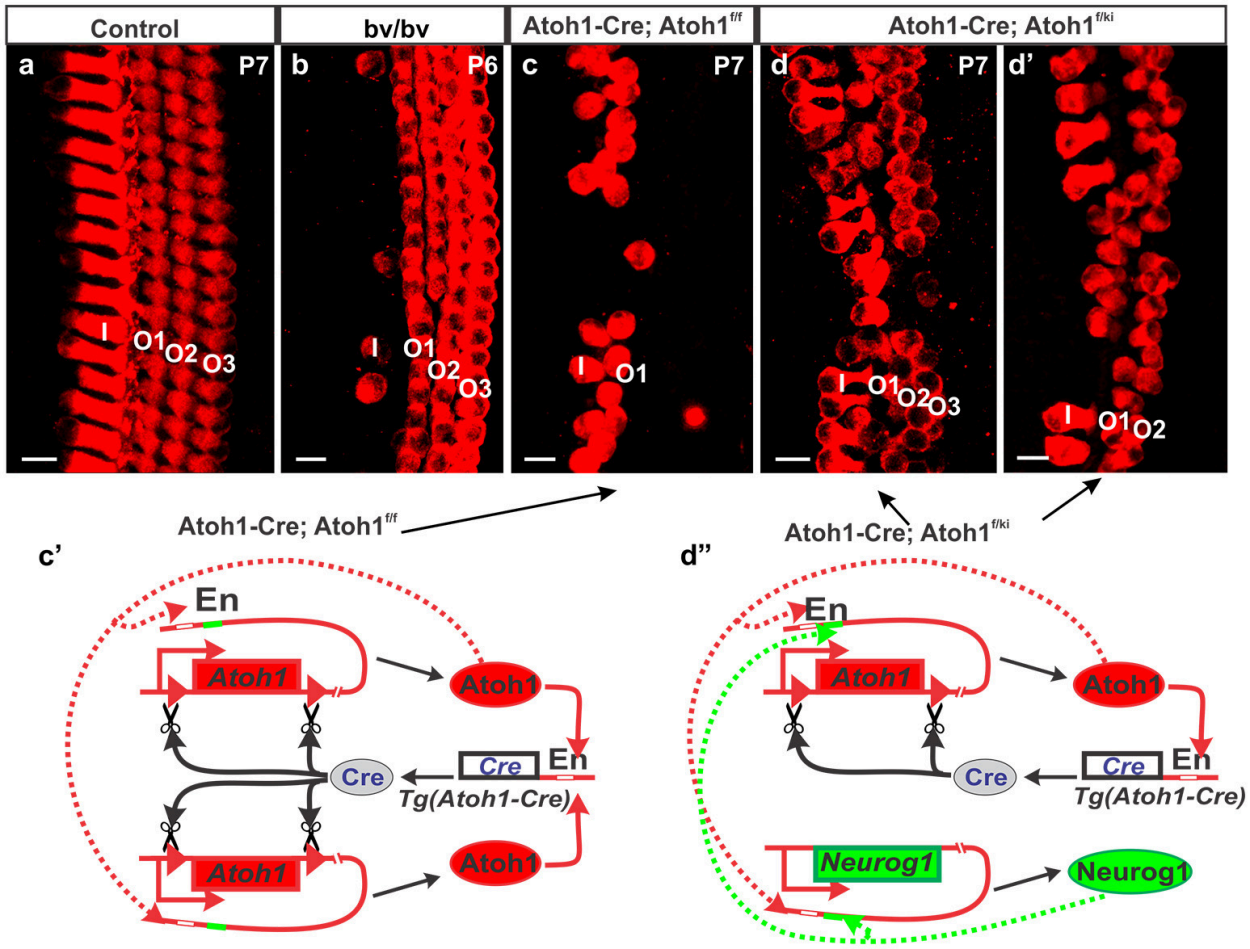
Different types of hair cell loss is shown for different mutant mice **(b**–**d)** compared to control mice **(a)**. Myo7a immunohistochemistry shows loss of most of the IHCs in Bronx-Waltzer (bv/bv) mutant mice with intact OHCs or occasional extra row of OHCs **(b)**, whereas deletion of *Atoh1* using self-terminating Atoh1-cre (*Atoh1-cre, Atoh1*^*f*/*f*^) results in loss of most of the IHCs as well as outer two rows of OHCs at P7 **(c)**. Misexpression of Neurog1 in Atoh1 locus in *Atoh1-cre, Atoh1*^*f*/*ki*^ mice shows massive rescue of both IHCs and OHCs numbers and alterations to the Atoh1 feedback loop **(c**′**)**, however the organization of HCs are not maintained **(d,d**′**)** even as the Atoh1 expression increases **(d**″**)**. Note that the enhancer element binding Atoh1 protein also has an adjacent eBox for Neurog1 protein that makes the potential interaction of both bHLH proteins difficult to assess. For a full review of the Atoh1 feedback loop see (Fritzsch and Elliott, 2017) Bar indicates 10 μm. Black arrows indicate evidence of the Atoh1 feedback loops in the Atoh1-CRE mutants.

Recent work has revealed local cellular variation in response to global gene manipulation. The longest known example is the Bronx-Waltzer mutation, a mutation of the differential splicing regulator protein Srrm4 (Nakano et al., 2012). This gene is expressed in all HCs, but the Bronx-Waltzer phenotype is characterized by IHCs loss that is for unknown reasons, variably penetrant with local sparing of some IHCs (Figure 2). Another example is the local variation of HC loss induced by the self-terminating system of *Atoh1-cre;Atoh1*^*ff*^, whereby a *Atoh1* enhancer element that binds the Atoh1 protein drives the expression of Cre (Matei et al., 2005) to recombine the floxed *Atoh1* gene (Pan et al., 2012). As expected from work on *Atoh1* null mutants (Bermingham et al., 1999; Fritzsch et al., 2005), HCs cannot fully differentiate despite an initial start toward differentiation. Surprisingly, however, many OHCs in the first row survive for up to 4 weeks, but only a few IHCs and OHCs in the second row survive, and then in a locally variable fashion (Figure 2). The variation in local cell response can be enhanced with another genetic manipulation whereby one *Atoh1* allele is replaced by *Neurog1 (Atoh1-cre; Atoh1*^*f*/*kiNeurog*1^*)*. This change rescues the vast majority of IHCs, and many more OHCs (Jahan et al., 2015b), but functionally, these mice are deaf (Tan et al., 2018), demonstrating that we must understand not only the formation of HCs and specific types, but also their complex physical interrelationship and interactions to form the stereotyped radial cellular assembly (Figure 2) along with the interplay between development and maintenance required for cell survival and function.

Local variation of HC types and surrounding supporting cell types can also be induced by eliminating *Neurod1*, a transcription factor (TF) that negatively regulates *Atoh1* expression levels. This genetic change leads only to local variation—some OHCs, only in the apex, express the IHC specific marker Fgf8 and develop into IHC-like cells (Jahan et al., 2010). This *Neurod1*-induced effect is due to unregulated increase in expression of *Atoh1* that is premature and enhanced in the apex OHC region (Jahan et al., 2015b), showing that intracellular feedback loops are an essential feature of local cell fate variations. A similar premature expression of Atoh1 follows loss of Neurog1 (Matei et al., 2005; Gálvez et al., 2017) a bHLH gene that is also regulated by Neurod1 (Jahan et al., 2010). The expression of Neurod1 is in turn regulated by Neurog1 (Ma et al., 1998) forming a complicated feedback loop with developing neurosensory cells of the ear (Fritzsch and Elliott, 2017). This is corroborated by local variations in response to ototoxic drugs (Sha et al., 2001), where cells in the base degenerate faster than in the apex with sharp lines of differential susceptibility along the length of the organ of Corti. Individual responses of HCs are also apparent in mice affecting the PCP pathway (Montcouquiol et al., 2003; Jones and Chen, 2008; Tarchini et al., 2016). The mechanism(s) underlying the differential effects of these HCs is in every case unclear, it is easy to speculate that transcriptional differences within the cells of these two regions are most likely playing a role but what differences exist and how much they very in nearby cells remains unknown.

## Unraveling the cellular transcriptome

Investigating the transcriptomes and translatomes of various cell types is at the heart of understanding the molecular biology of most cellular systems, and the auditory system is no exception (Kalisky et al., 2011; Hertzano and Elkon, 2012; Liu et al., 2014; Saliba et al., 2014; Schrauwen et al., 2014; Burns et al., 2015; Wilmarth et al., 2015; Ushakov et al., 2017). Traditionally, gene expression profiles have been investigated using probe-dependent methods (Kalisky et al., 2011), which require a high starting input, are low throughput, and time and cost ineffective (Saliba et al., 2014; Konry et al., 2016). For example, in 1994 Robertson et al. set out to profile gene expression in human fetal cochlea. After construction of the cDNA library, they employed subtractive hybridization, direct sequencing and manual analysis to sequence clones (Robertson et al., 1994). Their results yielded several genes later shown to be important for proper auditory function (Robertson et al., 1998). However it was not until microarrays were introduced in 2002 that cochlear gene expression really revealed its complexity (Chen and Corey, 2002; Cho et al., 2002). Microarray studies, in turn, were limited to the number of probes that could be manufactured on an array and miss novel splice variants of known transcripts and different single nucleotide polymorphisms.

**Figure 3:**
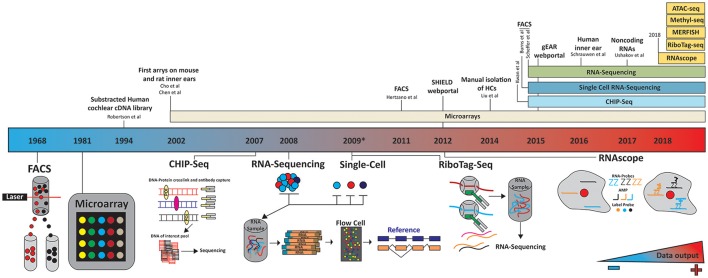
Timeline of technology evolution and its application to the inner ear. A panoply of technologies has been applied to the study of inner ear transcriptomes. These studies differed by the cell types investigated, isolation techniques (microdissection, FACS…) and subsequent processing platforms (microarrays, next generation sequencing). The future holds much promise as to the application of additional technologies such as RiboTag-seq and RNAscope to couple temporal and realtime spatial transcriptome profiling. Color gradient indicates the amount of data being generated, increasing blue to red. In 2012 and 2015 the SHIELD (http://shield.hms.harvard.edu) and gEAR (http://umgear.org/) databases were launched.

A decade later, single-cell RNA-sequencing is ushering in an era of “single cell-omics” and a new understanding of cochlear gene expression especially as it relates to temporal and spatial expression profiling (Nagalakshmi et al., 2008; Wu et al., 2017) (Figure 3). It is well established that the interplay of these two factors is essential to proper auditory development and function (Groves et al., 2013; Jahan et al., 2013; Swift and Coruzzi, 2017), and of the two, temporal expression profiling has always been the lower hanging fruit. For example, the first microarray studies of the inner ear compared gene expression across different developmental time points (Chen and Corey, 2002; Cho et al., 2002). In an attempt to provide a spatial context, studies also focused on specific regions of the cochlea, however the analysis was limited by the heterogeneity of cell types in every region of the cochlea (Cho et al., 2002; Cristobal et al., 2005; Nagalakshmi et al., 2008; Elkan-Miller et al., 2011; Jahan et al., 2013; Swift and Coruzzi, 2017; Wu et al., 2017).

This problem has been resolved to a great extent by using flow-cytometry and fluorescence activated cell sorter (FACS) to separate cell types (Figure 3). In 2011, Hertzano et al used this technology to separate specific cell populations by the cluster of differential antigens (CD proteins) they expressed (Hertzano et al., 2010, 2011). They identified five different cell types in the cochlea and vestibular systems at different developmental time points: HCs, sensory epithelial, mesenchymal, neuronal, and vascular endothelial. After sorting, expression profiles were generated using an array of more than 45,000 hybridization probes covering more than 33,000 genes and predicted genes to give the first spatial and temporal gene expression profiles of the cell populations in the inner ear. The authors used this approach to explore gene regulatory pathways in mutant mice, highlighting the diversity of genes expressed in each cell population, and providing to the scientific community a new resource with which to investigate gene regulation in the inner ear.

While the Hertzano dataset provides an unprecedented look into gene expression and patterning in the inner ear, there are some limitations, the most important of which are the limitations in cell sorting, as populations could only be grouped by CD-protein expression. For example, the cell cluster positive for CD326 and CD49f contain supporting cells, IHC, OHC, and the cells from the lesser and greater epithelial ridges (Hertzano et al., 2011). As an alternative, in 2014, Liu et al. characterized the transcriptomes of murine adult auditory IHCs and OHCs (Liu et al., 2014) by dissecting the organ of Corti, performing cell separation via an enzyme digestion, and manually picking and isolating each HC population (Liu et al., 2014). By pooling 2000 IHCs and OHCs, they generated transcriptome profiles using a GeneChip microarray (Figure 3). These results showed adult murine IHCs and OHCs are transcriptionally ~88% similar with ~1,500 genes significantly differentially expressed between the two HC types. Liu et al also highlighted the genes uniquely expressed in either OHCs or IHCs, roughly one-fourth of which are noncoding RNAs. While Liu provided us with the first look at the transcriptional profiles of the two HC populations, much knowledge was lost about gene expression over time and between neighboring cells. The profiling approach was a microarray, which masks much of the complexity associated with novel transcripts, RNAs and novel genes. The implementation of RNA-seq would be required to overcome these limitations.

It is well-established that the different cell populations of the inner ear are derived from a common embryonic cell type. While the identity of all players required to differentiate each cell population is not clear, several key molecules have been identified which are required to drive cell fate. Taking advantage of this knowledge, Scheffer et al. generated a reporter GFP-mouse under the HC specific Pou4f3 promoter (Scheffer et al., 2015). By FAC sorting, GFP positive vs. GFP negative cells, the authors were able to separate the cell population broadly into: HCs vs. non-HCs from the cochlea and utricle. Cells were harvested at four time points surrounding the mechanosensitivity development of HCs. The authors created pools of cells and performed nondirectional single end high throughput sequencing of 3′-tagged mRNA using an Illumina HiSeq. Using this approach, quantification of mRNA levels could be more accurately assessed (Asmann et al., 2009; Morrissy et al., 2009). While this study provided insight into gene expression surrounding mechanosensitivity development, it could not distinguish expression profiles between IHCs vs. OHC and all the non-GFP expressing cell populations. Furthermore, their approach is over representative of genes expressed in the OHC because they outnumber IHC about 3.2:1 (Jahan et al., 2015a). The authors also did not explore fully the unique opportunity to study the complexity and variety of RNA transcripts expressed in the ear, by focusing on only mRNA and the 3′-end of each gene (Steijger et al., 2013). Nonetheless, this study highlighted many unique opportunities RNA-Sequencing offers, to mine data that is not offered by traditional microarray.

Utilizing a similar approach Burns et al, used a triple transgenic reporter mice and FAC sorting of P1 mouse sensory epithelia to perform single cell bi-directional RNA-sequencing (Burns et al., 2015). The triple transgenic mice allowed the authors to analyze HC and supporting cells from both the utricle and cochlea. Similar to previous studies, the authors noted great transcriptional heterogeneity between each single cell, but homogeneity between cells of the same type. The authors identified several novel cell-type specific expressed genes and noted distant transcription profiles between the complimentary cell types of the auditory and vestibular system.

There are currently a whole host of technologies which can be used to unravel the transcriptome, translatome, and epigenome but their application to inner ear study has yet to be implemented (Figure 3). Undeniably, low sample input constitutes a challenge and a limitation for many of these technologies but as they evolve and advance, so will their sensitivity and versatility.

## Temporal variation in gene expression may drive local cell type variation

Above we outlined the current state of the art to analyze transcriptomes, mostly in differentiated cells. How much of a local variation possibly exits during the gene upregulation phase remains unclear as only cells that express markers to sort them can thus far be analyzed. No blind analysis of organ of Corti cells prior to the expression of HC specific markers has been completed, a necessary step to figure out the molecular means to regulate *Atoh1* expression in HCs that have exited the cell cycle for a variable time in a apex to base progression before the *Atoh1* upregulation happens mostly in a base to apex progression (Jahan et al., 2013). Here we explore principles of transcription regulation as well as various ways a cell can regulate the translation of message into protein to alter cell specific gene activation effects.

The central dogma describes the process of gene transcription and translation via the transcription of DNA into RNA and translation of RNA into protein. However, it does not address the imbalance or processing reduction at each step. That is, roughly 70% of the little over 3.6 billion nucleotides in the human genome are transcribed and only ~2% of the transcribed RNA is translated (Djebali et al., 2012). While the central dogma provides us a road map uniting DNA, RNA and protein; over the past two decades it has become clear this map is full of intersections, loops, tolls, and detours. This more refined map helps elucidate not only processing reduction, but expression in a temporal, spatial, and cell specific manner.

The inner ear provides a unique avenue to explore gene regulation in a spatial and temporal manner due to its extremely stereotyped pattern. Along its longitudinal axis, the cochlea exits the cell cycle from the apex to base, but if differentiates in the opposite manner (Figure 1). That is, the cells that first exit the cell cycle in the apex are the last to differentiate (Kopecky et al., 2013). Therefore, a HC at the base of the cochlea is more mature and reasonably assumed transcriptionally different than a HC in the middle or at the apex of the cochlea with others in between forming an as yet unclear gradient in their post-mitotic transcriptome that may underlie the well-known base to apex progression of HC loss under most conditions. This transcriptional gradient is not just limited longitudinally, but also radially. This axial gradient enhances in yet unclear ways the mosaic of radial patterning of cells comprising the cochlea governed by diffusible factors driving local variations (Jahan et al., 2015b; Munnamalai and Fekete, 2016) and planar cell polarity (Montcouquiol et al., 2003; Montcouquiol, 2006). But it must also give rise to the tonotopic diversity between longitudinal sections of the cochlea, and therefore between neighboring HCs as well. Thus, gene expression levels must be tightly regulated not only at the cell type level generating radial differences, but also between neighboring cells of the same type generating longitudinal differences (Figure 1).

Understanding how gene expression and regulation governs cellular diversity is a cornerstone of developmental biology (Peter and Davidson, 2011). Gene regulation can be broadly broken into: transcription, RNA-Splicing, mRNA-regulation, and translation regulation. While they can be conceived as separate entities realistically, each step is simultaneously co-existing and functioning in unison which includes elaborate feedback regulatory loops on their own expression regulation (Figure 2) as well as other genes (Fritzsch and Elliott, 2017) that can be revealed in the local cell variations after global gene manipulations.

## Transcription

The regulation of transcription and the elements involved have come more into the spotlight as our understanding of the genome composition become clearer and technologies to study these elements have become more accessible (Cattoni et al., 2015). There are many ultraconserved DNA gene regulatory elements including: enhancers, silencers, insulators and promoters. Briefly, enhancers and silencer regulate gene expression positively and negatively, respectively. Insulators, help regulate the enhancers and silencers and often mark the boundary of each gene regulatory element (Vietri Rudan and Hadjur, 2015; Bonev and Cavalli, 2016). Those ultraconserved regions have long been recognized but only recent gene manipulations using simultaneous excision of several of these elements reveal some functional effects (Spurrell et al., 2016; Dickel et al., 2018). Unfortunately, the roles of these ultraconserved elements are largely unexplored in the development of the cochlea. However, there are several examples in both humans and mice where enhancers have been shown to be of functional importance (Rodriguez-Paris and Schrijver, 2009; Wilch et al., 2010; Masuda et al., 2017; Johnson et al., 2018). Extrapolating what is known thus far indicates that they could be of potential importance for subtle local variations of the same cell type within the cochlea.

Promoters and their corresponding binding partners, TFs, have been subject to detailed exploration in the inner ear. Indeed, a cocktail of TFs have been identified and characterized which drive cellular fate in the inner ear and are required for proper inner ear development (Fritzsch et al., 2010; Li S. et al., 2016). Although it is well established TFs are key regulators in cellular fate, only recently has the landscape of TFs expressed between different cell types in the ear been revealed (Li et al., 2016b). The data shows both HC population express >1,500 transcription factors in common and only a fraction (73 in IHCs) and (13 in OHCs) that are differentially expressed (Li et al., 2016b). This suggests that a few tightly regulated TFs are ultimately responsible for cellular identity, differentiation and function of the HC populations. It is easy to speculate that the differentially expressed TFs are the TFs that drive HC specific gene expression such as *OTOF* in the IHCs and *SLC26A5* in the OHCs. Elucidating the promoters these differentially expressed TFs bind, will further help unravel the transcriptional architecture defining each HC population.

Mutant animal models have played a vital part in understanding the regulatory networks of many TFs. Besides, the pathomorphological consequences that can be elucidated from mutant models, gene expression profiling can be used to understand the network of genes regulated by a TF (Hertzano et al., 2004, 2011; Sanchez-Calderon et al., 2010; Sajan et al., 2011; Cai et al., 2015; Elkon et al., 2015; Li S. et al., 2016; Matern et al., 2017). For example, in the late 1990's the transcription factor POU4F3 was identified as the gene underlying DFNA15 deafness in humans (Vahava et al., 1998) and demonstrated to be fundamental for proper HC development in the murine cochlea (Erkman et al., 1996). These findings sparked the pursuit to elucidate the role of POU4F3 in inner ear development. It became clear that Pou4f3 is an early regulator of HC development and acts as a regulator for other transcription factors such as *Gfi1, Lhx3, BDNF*, and *NT-3* (Xiang et al., 2003; Hertzano et al., 2004, 2007). Unraveling the hierarchy of Pou4f3 network has provided further insights into HC-type transcriptional networks. *Lhx3*, another HC specific TF is regulated by Pou4f3 in auditory HCs but not vestibular HCs, where it is also expressed. Studying the role of Pou4f3, not only revealed key regulators in HC development, maturation and function but also provided insight into topologically organizing neuronal innervation to HCs (Xiang et al., 2003). While these studies are invaluable, they require a mutant animal model with its strength and limitations. Exploring the binding motif computationally is another option when mutant models are not available. While mutant models can highlight increases or decreases in gene expression, it is often unclear which regulatory element they are acting on for a given gene. Computational methods can typically resolve the why by providing predictions of genomic location of a given TF binding (Jayaram et al., 2016). Coupling the mutant expression data with the computational predictions for a given TF could unravel how these TF regulate gene expression outside promoters and provide more insight into gene regulation in the inner ear. Experimentally, techniques such as ChIP-seq (Johnson et al., 2007) (chromatin immunoprecipitation coupled with massively parallel sequencing) can be used to investigate TF binding sites. ChIP-seq has been used to investigate promoter binding of C-MYC and SOX2 in immortalized multipotent otic progenitor cells (Kwan et al., 2015), it has yet to be used on differentiated cell types in the ear. Although studying the transcriptomes of knockout models targeting TFs has given great insight into the genes regulated by specific TFs, it is not a practical or viable approach. Rather ChIP-seq offers a high throughput method to evaluate transcriptional networks.

## Splicing governing expression and function

Alternative splicing, the assembly of mRNA from the RNA transcript, is an essential process regulating gene expression. By alternative splicing, humans can turn the ~20,000 protein coding genes into >290,000 peptides (Kim et al., 2014). Most of these proteins show high similarity, only differing slightly in domains, resulting in modulation of protein function. Alternative splicing is also a regulator of gene expression, creating spliced transcripts containing different exons in a spatial and temporal manner by giving rise to prematurely truncated open reading frames (ORFs), affecting mRNA stability, and targeting by microRNAs, or even translation efficiency. Since the core spliceosome is required for proper splicing, cell type specific *trans* splicing factors (both protein and non-coding RNAs) are the regulators of cell type specific alternative splicing (Breitbart and Nadal-Ginard, 1987; Singh et al., 2015).

In the inner ear, disruption in alternative-splicing has been shown to cause hearing loss. Alterations to the *Srrm4* gene, an alternative-splicing regulator, results in hearing and balance impairment (Nakano et al., 2012). By comparing exon composition of RNAs between Srrm4 mutants and wildtype mice the authors identified a group of RNAs that were dependent on Srrm4 function for proper mRNA formation. Comparison of all affected transcripts revealed a common motif, which recruits Srrm4 for participation in splicing. Similarly, disrupting the splicing factor Sfswap in mice, causes vestibular and cochlear defects most likely through gene disruption of the Notch signaling pathway (Moayedi et al., 2014). More recently, mutations in epithelial splicing-regulatory protein *ESRP1* have been linked to deafness in humans and inner ear developmental defects in mice (Rohacek et al., 2017), as a consequence of more than 500 mis-splicing events.

The NOVA family of splicing proteins have recently been shown to play a critical role in inner ear efferent innervation through regulating pathfinding properties of efferent axons (Saito et al., 2016). Loss of NOVA1 alone does not change efferent innervation to the cochlea, but the NOVA1 alone is not sufficient to maintain function. In contrast, loss of NOVA2 results in a decrease of innervation. When both NOVA1 and 2 are removed the innervation stalls when the efferent neurons reach the vestibular ganglion neurons (Saito et al., 2016). The difference in resulting physiological consequences from the loss of either or both of these splicing factors to the ear, might also be explained by the cell types in the ear that express NOVA1 and 2. At the mRNA level, NOVA2 is highly expressed in the HCs compared to NOVA1 which is highly expressed non-sensory cell types^1^ Oblation of NOVA1 or NOVA2 specifically in the ear would reveal which inner ear RNAs are regulated by which NOVA family member and may shine light on molecules important in guiding efferent neuron innervation to the cochlea.

Simply analyzing the large number of genes involved in differential splicing could reveal several more important proteins that regulate local translation variation and thus cryptic cell subtypes that can only be revealed through mutations or sophisticated unbiased transcriptomics.

While inner ear specific splicing factors are still greatly under-investigated, it has been hypothesized that the cochlea tonotopic gradient may be governed by it (Xu et al., 2007; Miranda-Rottmann et al., 2010; Sakai et al., 2011). The pathomorphological and phenotypic spectrum associated with many genes in the inner ear may also be controlled in such a manner. Genes that have alternative splicing and a variable deafness phenotype provide a unique opportunity to further elucidate inner ear specific splicing factors. For example, while both alternatively spliced isoforms of the *Whirlin* gene are required for proper hearing, the phenotype associated with defects in *Whrn* are dependent on the isoform(s) altered (Ebrahim et al., 2016). The tip-link forming genes *Cdh23* and *Pcdh15* (Kazmierczak et al., 2007) are also examples of alternative-splicing in a spatial and temporal manner, respectively. *Cdh23*, exists as two splice variants. While the short isoform is widely expressed, the long isoform is only expressed in the inner ear (Siemens et al., 2004). Unique splice forms of *Pcdh15* are required in a temporal fashion for the proper function in HCs (Webb et al., 2011; Pepermans et al., 2014; Pepermans and Petit, 2015). While these are just a few examples, several other genes also undergo similar alternative-splicing events, giving rise to unique and essential peptides required for proper auditory development and function (Ouyang et al., 2002; Michalski et al., 2009; Ben Rebeh et al., 2010; Khateb et al., 2012).

## mRNA regulation

**Figure 4:**
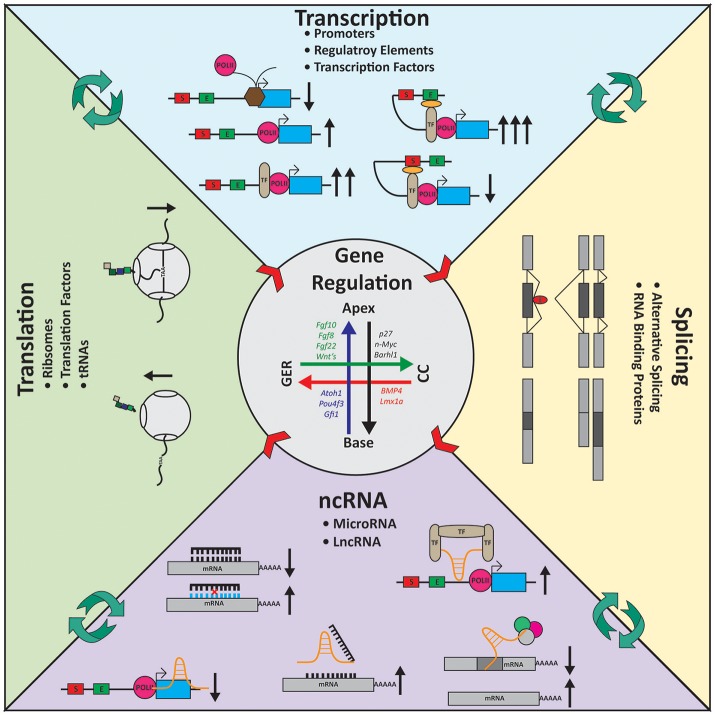
Gene regulation in the inner ear. Expression gradients are shown for several genes in the inner ear longitudinally from the apex to the base and radially from the Greater Epithelial Ridge (GER) to the Claudius cells (CC). Transcription: For transcription to occur polymerase II (POLII) needs to be able to bind the promoter of the gene. Transcription factors (TF) helps regulate transcription activity. TFs can bind the POLII machinery and increase transcription or can coordinate regulatory elements such as enhancers (E) or silencers (S) to modulate transcription. Splicing: Alternative splicing gives rise to a diverse group of mRNA molecules containing unique sequences. RNA binding proteins (RBP, red) help coordinate which exons are included or excluded from transcripts. Cell-type specific splicing factors ensure the essential transcripts required by the cells are being properly assembled. Noncoding RNA (ncRNA): ncRNAs work on many levels to control gene expression. MicroRNAs (black) typically bind 3′ untranslated regions of genes and degrade the mRNA via the RISC-pathway. Long noncoding RNAs (lncRNAs, orange) are multi-faceted. They can block POLII from elongation; act as a decoy target for microRNAs; coordinate/scaffold TFs or bind mRNA directly. Translation: Ribosomes (gray) are responsible for translation. Intrinsic mechanisms such as traffic-jams, the holding on the mRNA molecule by reading through the stop codon, and translation efficiency alter expression at the protein level. Center arrows represent the gradient of gene expression laterally and longitudinally (also see Figure 1). Black arrows indicate the consequence (increase or decrease) at each stage of regulation based on the molecular action depicted.

The majority of RNAs transcribed lack an open reading frame (ORF) required for translation, these are referred to as non-coding RNAs (ncRNAs; Figure 4). While there are a whole host of non-coding RNAs, ranging in size and varying in function (St Laurent et al., 2015), when it comes to gene regulation microRNAs (miRNAs) and long ncRNAs (lncRNAs) are at center stage (Bushati and Cohen, 2007; Batista and Chang, 2013; Kung et al., 2013; Cech and Steitz, 2014). While microRNAs have been in the spotlight since the late 1990's (Lee et al., 1993; Wightman et al., 1993), lncRNAs and their multifaceted functions are only more recently becoming widely studied (Nagano and Fraser, 2011; Spitale et al., 2011; Wang and Chang, 2011; Wapinski and Chang, 2011; Wutz, 2011; Bonasio and Shiekhattar, 2014; Sun et al., 2017).

miRNAs are a class of small ncRNAs that can belong to the most conserved short DNA stretches across a vast array of phyla (Pierce et al., 2008). A majority of miRNAs are transcribed in clusters or as part of introns of other genes, later removed and processed via the Drosha-Dicer pathway into small ~22 nucleotides RNAs (Sand, 2014; Ratnadiwakara et al., 2017; Song and Rossi, 2017). After maturation, miRNAs bind to their mRNA targets 3′ untranslated region (UTR) determined by their 7 base pair seed region (Sand, 2014). The binding of its target acts as a signal and targets the bound mRNA for degradation through the RNA interference pathway (Guo et al., 2010). Many miRNAs are transcribed as part of larger RNAs via polymerase II, and as such their expression is tightly regulated under RNA they are transcribed with.

In the ear, microRNAs and their targets have been at the heart of many studies and excellent reviews for more than a decade (Wienholds et al., 2005; Weston et al., 2006, 2011; Pierce et al., 2008; Soukup et al., 2009; Patel and Hu, 2012; Li et al., 2016a; Riccardi et al., 2016; Wang Y. et al., 2016; Ebeid et al., 2017; Huang et al., 2017; Hu et al., 2018). In this time more than 400 miRNAs have been identified through traditional microarray studies (Weston et al., 2006; Wang et al., 2010; Elkan-Miller et al., 2011; Hertzano et al., 2011; Steijger et al., 2013; Zhang et al., 2013, 2014) and RNA-Sequencing (Rudnicki et al., 2014; Riccardi et al., 2016), but only a handful has been characterized. The biogenesis of miRNAs has been shown to be fundamental in inner ear and sensory development by the ablation of *Dicer1* (Soukup et al., 2009; Kersigo et al., 2011). Remarkably, ablating *Dicer1* around E12.5-E14.5 using either the Atoh1:Cre or Pou4f3:Cre, does not affect cochlea or HC development (Friedman et al., 2009; Weston et al., 2011), highlighting the stability of miRNAs (Figure 4). However, continued renewal of miRNAs and the expression of new miRNAs is essential for HC maintenance. Intriguing, both Cre-lines produce the same longitudinal variably in HC death, with the more severe loss at the base than the apex. This gradient in HC death strongly indicates there is most likely an axial gradient of miRNA expression in the cochlea, used as a modulator of gene expression. Given our evolving knowledge about the transcriptomics of specific cell populations in the inner ear, cell-type specific *Dicer1* conditional knockouts would further help elucidate the roles of miRNAs in the inner ear. Alternatively, with the rapid advancements in the CRISPR-Cas9 technology, deriving miRNA-specific mutants should become more easily doable. It should be noted that the delay in depletion of miRNAs after Dicer conditional deletion can show local and cell type specific variations making the interpretation of this approach very difficult and require supplementing the investigations on miRNA function through targeted deletion of one or more miRNAs (Kersigo et al., 2011; Weston et al., 2011).

An area of regulation that has been greatly ignored in the inner ear is long non-coding RNAs. LncRNAs are broadly defined as RNA molecules >200 nucleotides, lacking an ORF. Many lncRNAs contain multiple exons and are subjected to alternative splicing and undergo the same post-translational modification as protein coding transcripts (Guttman et al., 2009). LncRNAs can be characterized by: genomic location (intergenic, gene-overlapping, divergent or antisense), function (signal, decoy/molecular sponge, scaffold, guide, or enhancer), regulatory level of effect (transcription, splicing, mRNA stability or translation) and subcellular localization (nucleus, cytoplasm or extracellular) (Devaux et al., 2015; Bär et al., 2016). Since lncRNAs are transcribed by polymerase II (Djebali et al., 2012), they are subjected to the same temporal and spatial transcriptional regulation as all other RNAs. LncRNAs can directly influence gene regulation or acts as regulators to the regulators of regulation by sequestering miRNA (Figure 4).

Transcriptome studies of inner ear tissues have started to unravel the expression profiles of lncRNAs in the murine inner ear (Liu et al., 2014; Ushakov et al., 2017). Ushakov et al. revealed that in the mouse inner ear lncRNAs are plentiful, expressed spatially and temporally and >20 lncRNAs which show potential to influence genes already known to be important for inner ear function in mice and humans (Ushakov et al., 2017). The authors identified several interesting lncRNAs. Firstly, the authors described a lncRNA for mir96. As discussed above, mircoRNAs act as fine tuners of gene regulation. The authors show lnc-mir96 is differentially expressed between the cochlea and vestibule and varies over time. LncRNAs have been described to act as decoy targets for miRNA binding (Rani et al., 2016) allowing for an increase of translation of the miRNA native targets. While characterization studies are needed, it is hard not to speculate that lnc-mir96 might regulating mir96 or the highly conserved mir183 triad. Another interesting finding is the lncRNA, transcribed inside the BMP4 gene. It is well established that BMPs are found in a sharply decreasing gradient from the OSCs (Claudius cells) to the IPCs (Pan et al., 2012; Munnamalai and Fekete, 2016). This lncRNA could function as a regulatory molecule fine tuning of this gradient. Finally, a lncRNA was identified upstream of the *Gjb2* gene. In humans it has been shown that *GJB2* expression is regulated by a cis-regulatory element upstream of *GJB2* (Rodriguez-Paris and Schrijver, 2009; Wilch et al., 2010). Many lncRNAs act as enhancers (Ørom et al., 2010; Kowalczyk et al., 2012), given its proximity then lncRNA found upstream of *Gjb2* in the Ushakov study may act similarly to the *GJB2* regulatory element in humans. While this remain speculative, with the increasing advancement in technologies used to study lncRNAs, these functions should be elicited in the near future.

While large transcriptome studies are undoubtedly important, in general lncRNAs are not highly conserved between species (Ponjavic et al., 2007) in stark contrast to the extreme conservation of some ear specific miRNAs (Pierce et al., 2008). This lack of conservation and the lack of human inner ear material to profile them make it difficult to draw conclusions about lncRNAs found in the murine ear. In 2014, Schrauwen and colleagues (Schrauwen et al., 2014) profiled the transcriptomes from human cochlea, saccule and utricle. The authors identified more than 7,000 lncRNAs with more than 250 differentially expressed in the inner ear. The authors also highlighted the use of unique spliced transcripts specific to the inner ear vs. other tissues and preferential splicing between the cochlea, saccule and utricle (Schrauwen et al., 2014). These RNA-Seq studies both in mice and humans, continually highlight the complexity of gene regulation in the inner ear that remains to be explored in its functional significance.

An interesting subclass of lncRNAs is pseudogenes (for a comprehensive review of pseudogenes see Vanin, 1985; Balakirev and Ayala, 2003). Some pseudogenes can escape nonsense mediated decay and act as regulators of gene expression (Kalyana-Sundaram et al., 2012; Lappalainen et al., 2013; Tang et al., 2017), though the mechanism and their mode of action is poorly understood. While studying the human cochlea transcriptome, Schrauwen et al. detected >150 pseudogenes of which 50% were differentially expressed between the different inner ear tissue types (Schrauwen et al., 2014). In humans, the *STRC* and *OTOA* genes both have pseudogenes, of unknown function. Large genomic conversions between the parent *STRC* gene and its pseudogene have been shown to result in deafness in humans. The temporal and spatial expression of the *STRC-pseudogene* and the *OTOA-pseudogene* has yet to be elucidated. The consequence of ablating these pseudogenes is currently unclear, but unraveling their role may open up new avenues for therapeutics (Roberts and Morris, 2013).

In summary, the formation of mRNA molecules and more broadly gene expression is complex and cell type specific. Although there is temporal and spatial data showing differences in gene expression in the cochlea, these studies are too broad to guide cell type and subtype specific regulation. Applications such as RNAscope (Wang et al., 2012), multiplex error-robust FISH (MERFFISH) (Chen et al., 2015), and spatial transcriptomics (Stahl et al., 2016) allow for quantifiable single cell resolution of gene expression *in vivo*. These applications to the cochlea are needed to unravel the subtle but critical quantitative changes in gene expression along both axis of the cochlea and in adjacent cells to detail how local gene expression variations are embedded into systematic, longitudinal changes.

## Translation regulation

The final part of the central dogma is the translation of RNA into protein. The three topics discussed above all impact this final step. Similar to the three other steps described, it is becoming increasingly clear that translation regulation is highly coordinated and complex and evolved an ever growing number of players (Shi and Barna, 2015). Like mRNA is quantitatively expressed between cells, protein is as well. Interestingly, the amount of mRNA only plays a partial role in how much gets translated (Schwanhüusser et al., 2011). That is, levels of mRNA do not always correlate to the amount of protein being transcribed, but rather are heavily dependent on the translation and release efficiency of the ribosomes (Gebauer and Hentze, 2004; Sonenberg and Hinnebusch, 2009; Schwanhüusser et al., 2011; Yordanova et al., 2018) and the half-life of the protein itself (Sandoval et al., 2013; Stevens and Brown, 2013). One interesting trend which could be more easily exploited to study translation regulation in the inner ear is protein-turnover/half-lives (Figure 4). Many extracellular matrix proteins, like collagens, have long half-lives (Verzijl et al., 2000; Terjung, 2010). A diverse group of collagens are known to be important for proper auditory function; focusing studies on these molecules might provide great insight into regulatory mechanisms of translation in the inner ear. Furthermore, exploiting applications such as RiboTag-Seq (Sanz et al., 2009) or real-time in situ measurement of translation (Wang C. et al., 2016) would inevitably unravel which mRNAs are being translated and ultimately contribute to the translatome itself. This knowledge will highlight which peptides exhibit differential temporal and spatial expression.

## Lessons learned: how to regulate local variability to generate specific hair cell subtypes in specific areas to functionally restore hearing in deaf people

Hearing loss and impairment is recognized by the WHO as the most frequent ailment of our global population with predicted increase to around 1 billion people affected by 2050 as the world's population ages. Of those, several hundred million people likely suffer from neurosensory hearing loss and impairment requiring either a cochlear implant, pharmacological or molecular therapies to restore hearing (Zine et al., 2000).

Pharmacological intervention to decrease or slow cell death in the inner ear has been slow and limited in success. Currently, we still lack the knowledge of which genes and ultimately pathways are being activated after cell stress or damage. One promising pathway for pharmacological intervention is the JNK/c-Jun signal pathway that is activated in HCs after trauma (Pirvola et al., 2000). It has been recently shown that by blocking the c-Jun pathway, through genetic manipulation or pharmacologically, HCs exposed to stress and damage survive at a greater rate (Anttonen et al., 2016). Further exploration into c-Jun pathway may unravel other proteins for targeting. More broadly, a transcriptomic level, understanding gene expression between “healthy” cells and those that have been damaged will undoubtedly illuminate a broader group of potential targets for intervention.

Molecular hearing restoration through molecular supporting cell conversion thus far has only achieved minor local effects mostly in postnatal animals and only shortly after removing HCs by various means lasting for a limited time. This paucity of success in the organ of Corti contrasts with progress in restoring the much simpler mosaic of vestibular sensory epithelia in adult mammals (Bucks et al., 2017). Importantly, current attempts for hearing restoration provide no clear path forward how to overcome the roadblocks facing molecular hearing restoration of the organ of Corti (Bucks et al., 2017). More recently it was for example found that combined expression of *Atoh1* with its apparent downstream factor *Pou4f3* leads to more effective HC differentiation *in vitro* compared to each factor alone (Costa et al., 2015). It is possible that late in development unknown feedback loops limit the effective drive of *Pou4f3* by *Atoh1* and thus require the additional and simultaneous expression of additional factors. Given that the self-regulated *Atoh1* upregulation is limited by *Neurod1* (Kopecky et al., 2013), one possible way to achieve more profound self-regulatory effects of *Atoh1* would be the expression of *Atoh1* combined with inhibition of *Neurod1*. Even co-expression of *Atoh1* with HC specific miRNAs (Weston et al., 2011) might boost transformation of HC precursors into stably differentiated HCs, something that has not been achieved thus far. Combining specific miRNAs with TFs greatly enhance the neuronal transdifferentiation process (Xue et al., 2013). In fact, the champion of regeneration among vertebrates, the salamanders, seemingly use multiplication of miRNAs for this ability (Elewa et al., 2017; Nowoshilow et al., 2018). Consistent with this is most recent work that suggests enhanced HC differentiation using miRNAs (Kim et al., 2018).

Once the molecular basis for radial and longitudinal expression variation has been more full explored, techniques need to be developed that allow locally variable regulation of multiple transcripts to mimic known pattern of expression variations. This could enable to move current attempts toward hearing regeneration forward to generate local variations of HC types appropriate to restore the exquisite tuning properties of the organ of Corti.

Understanding better the transcriptome landscape of early differentiation organ of Corti cells (Durruthy-Durruthy et al., 2014) at several critical steps in a regional specific way could provide a better guidance of these efforts also toward a more lasting effect compared to current attempts only able to generate transient HCs. Understanding not only how to make a specific radial cell type but how to fine tune its local variation (Figure 1) of gene expression (Figure 4) to drive the tuning properties in a specific location seems to be beyond what can be achieved in the immediate future. Such regulation will ultimately be required to guide full functional restoration *in vivo* that exceeds current technical abilities provided by the cochlear implant. Such more precise local regulation of cell fate could allow tonotopic hearing whereby adjacent IHCs are tuned to distinct frequencies to allow absolute pitch hearing over a dynamic range.

## Author contributions

KB, HA, and BF: layout of the review, written part of the review, contributed images and proof read the review. RS and IJ: contributed images, added text, read, and approved the manuscript.

### Conflict of interest statement

The authors declare that the research was conducted in the absence of any commercial or financial relationships that could be construed as a potential conflict of interest.

## Footnotes

^1^gEAR—gene Expression Analysis Resource web portal. Available online at: https://umgear.org/
